# ZIPK collaborates with STAT5A in p53-mediated ROS accumulation in hyperglycemia-induced vascular injury: ZIPK collaborates with STAT5A in hyperglycemia-induced ROS accumulation

**DOI:** 10.3724/abbs.2024120

**Published:** 2024-07-19

**Authors:** Qichao Wu, Tingting Xie, Chang Fu, Chenyu Sun, Yan Ma, Zhengzhe Huang, Jiao Yang, Xiaoxiao Li, Wenqian Li, Changhong Miao

**Affiliations:** 1 Department of Anesthesiology Zhongshan Hospital Fudan University Shanghai 200031 China; 2 Shanghai Key Laboratory of Perioperative Stress and Protection Shanghai 200031 China; 3 Department of Anesthesiology Zhongshan Hospital (Xiamen) Fudan University Xiamen 361015 China; 4 Department of Anesthesiology Fudan University Shanghai Cancer Center Shanghai 200031 China; 5 Department of Oncology Shanghai Medical College Fudan University Shanghai 200031 China

**Keywords:** ZIPK, STAT5A, p53, ROS accumulation

## Abstract

In this study we investigate the role of Zipper-interacting protein kinase (ZIPK) in high glucose-induced vascular injury, focusing on its interaction with STAT5A and its effects on p53 and inducible nitric oxide synthase (NOS2) expression. Human umbilical vein endothelial cells (HUVECs) are cultured under normal (5 mM) and high (25 mM) glucose conditions. Protein and gene expression levels are assessed by western blot analysis and qPCR respectively, while ROS levels are measured via flow cytometry. ZIPK expression is manipulated using overexpression plasmids, siRNAs, and shRNAs. The effects of the ZIPK inhibitor TC-DAPK6 are evaluated in a diabetic rat model. Our results show that high glucose significantly upregulates ZIPK, STAT5A, p53, and NOS2 expressions in HUVECs, thus increasing oxidative stress. Silencing of *STAT5A* reduces p53 and NOS2 expressions and reactive oxygen species (ROS) accumulation. ZIPK is essential for high glucose-induced p53 expression and ROS accumulation, while silencing of *ZIPK* reverses these effects. Overexpression of ZIPK combined with STAT5A silencing attenuates glucose-induced alterations in p53 and NOS2 expression, thereby preventing cell damage. Coimmunoprecipitation reveals a direct interaction between ZIPK and STAT5A in the nucleus under high-glucose condition. In diabetic rats, TC-DAPK6 treatment significantly decreases ZIPK, p53, and NOS2 expressions. Our findings suggest that ZIPK plays a critical role in high glucose-induced vascular injury via STAT5A-mediated pathways, proposing that ZIPK is a potential therapeutic target for diabetic vascular complications.

## Introduction

Diabetes mellitus (DM) is a chronic metabolic disorder characterized by hyperglycemia resulted from defects in insulin secretion, insulin action, or both. According to the International Diabetes Federation (IDF), approximately 463 million adults were living with diabetes in 2019, and this number is projected to increase to 700 million by 2045 [1]. This disease is associated with significant morbidity and mortality, with cardiovascular complications being the leading cause of death among diabetic patients [2]. Despite advancements in diabetes management, including lifestyle modifications, oral hypoglycemic agents, and insulin therapy, the prevention and treatment of diabetes-related vascular complications remain significant challenges [ 3, 4] .

Vascular complications in diabetes are primarily driven by endothelial dysfunction, which is exacerbated by hyperglycemia-induced oxidative stress and inflammation [ 5, 6]. Endothelial cells, such as human umbilical vein endothelial cells (HUVECs), play a crucial role in maintaining vascular homeostasis. Hyperglycemia induces the production of reactive oxygen species (ROS) in endothelial cells, leading to cellular damage and apoptosis [ 7, 8]. Furthermore, the dysregulation of various signaling pathways, including those involving protein kinases, is implicated in the pathogenesis of diabetic vascular complications [ 9, 10].

Zipper-interacting protein kinase (ZIPK), also known as DAPK3, is a serine/threonine kinase involved in various cellular processes, including apoptosis, autophagy, and cytoskeletal organization [ 11, 12]. Recent studies have suggested that ZIPK may regulate endothelial cell function and vascular integrity [ 13, 14]. For instance, ZIPK interacts with and modulates the activity of signal transducer and activator of transcription 5A (STAT5A), a transcription factor involved in cell growth and survival [ 15, 16]. Additionally, ZIPK regulates p53, a key mediator of cellular stress responses, and nitric oxide synthase 2 (NOS2), an enzyme involved in nitric oxide production and a critical regulator of vascular tone and homeostasis [ 17, 18].

Given the potential role of ZIPK in regulating endothelial cell function and its interaction with key signaling molecules implicated in diabetic vascular complications, this study investigated the role of ZIPK in high glucose-induced vascular damage. Specifically, we examined the effects of high glucose on the expressions of ZIPK and its downstream targets STAT5A, p53, and NOS2 in HUVECs, as well as the impact of ZIPK modulation on oxidative stress and vascular integrity.

In the present study, HUVECs were cultured under normal glucose (5 mM) and high glucose (25 mM) conditions for 6 days to mimic the diabetic environment. Protein expression levels were assessed by western blot analysis, and gene expression levels were measured using quantitative real-time PCR (qPCR). ROS levels were evaluated using flow cytometry, and protein interactions were analyzed through immunoprecipitation and immunofluorescence techniques. Additionally, a diabetic rat model was used to investigate the effects of ZIPK inhibition on glomerular vascular damage. Our findings show that ZIPK plays a critical role in high glucose-induced vascular injury via STAT5A-mediated pathways, proposing that ZIPK is a potential therapeutic target for diabetic vascular complications.

## Materials and Methods

### Cell culture

Human umbilical vein endothelial cells (HUVECs) were obtained from Procell (Wuhan, China). The cells were cultured in DMEM (Clonetics, Lonza, Switzerland) supplemented with 10% certified heat-inactivated fetal bovine serum (Yeasen Biotechnology, Shanghai, China), 1% penicillin (100 U/mL; Yeasen Biotechnology), and 1% streptomycin (100 μg/mL; Yeasen Biotechnology) at 37°C in a 5% CO _2_ humidified atmosphere. Cells cultured for 6 days in normal glucose (5 mM) DMEM served as the control group (Con), while those cultured for 6 days in high glucose (25 mM) DMEM constituted the high glucose group (HG).

### Western blot analysis

Cell lysis was performed using RIPA lysis buffer (Beyotime Biotechnology, Shanghai, China). The concentration of total cellular protein extracts was quantified using a BCA assay kit (Thermo Fisher Scientific, Waltham, USA). Equal amounts of protein were separated by 8%‒15% SDS-PAGE and transferred onto a polyvinylidene fluoride (PVDF) membrane (Millipore, Billerica, USA). The membrane was blocked with 5% skimmed milk and then incubated with specific primary antibodies ( Table 1) overnight at 4°C. The following day, the membrane was incubated with the HRP-conjugated secondary antibodies ( Table 1) at room temperature for 1 h. Protein detection was performed using an enhanced chemiluminescence (ECL) system (Shanghai Epizyme Biomedical Technology Co., Ltd., Shanghai, China). The band intensities were quantified using ImageJ software (National Institutes of Health, Bethesda, USA). β-Actin was used as an internal standard.

**Table:** **Table 1** Primary and secondary antibodies used in this study

Antibody	Catalog number	Dilution ratio	Source
β-actin	81115-1-RR	1:1000	Proteintech
ZIPK	30656-1-AP	1:1000	Proteintech
STAT5A	66459-1-Ig	1:1000	Proteintech
P53	60283-2-Ig	1:1000	Proteintech
NOS2	80517-1-RR	1:1000	Proteintech
HRP-conjugated goat anti-rat IgG	A0192	1:2000	Beyotime Biotechnology
HRP-conjugated goat anti-Rat IgG	A0277	1:2000	Beyotime Biotechnology

### Quantitative real-time PCR (qPCR)

Total RNA was isolated using Trizol reagent (Tiangen, Beijing, China). A total of 1 μg total RNA was reversely transcribed into complementary DNA (cDNA) with PrimeScript RT reagent kit (TaKaRa, Shiga, Japan) using oligo(dT) primer at 42°C for 1 h, and 2 μL the reverse transcription reaction mix was amplified by PCR with denaturation at 95°C for 2 min, followed by 50 cycles of 95°C for 30 s, 55°C for 30 s, and 72°C for 1 min. Then, qRT-PCR assay was carried out using SYBR Green PCR Kit (TaKaRa) with the 7300 Real-Time PCR System (Applied Biosystems, Carlsbad, USA). *β-Actin* was used as an internal control. The sequences of the qPCR primers used are listed in Table 2. Relative gene expression levels were calculated using the 2 ^‒ΔΔCT^ method, with data presented as fold changes relative to the control, which was set as 1.

**Table:** **Table 2** Sequence of primers used for real-time RT-PCR

Gene	Primer sequence (5′→3′)
*β*- *Actin*	F: CTTCCAGCCTTCCTTCCTGG
	R: GAGCCACCAATCCACACAGA
*ZIPK*	F: ATTTGTACCGGAGGTTCTCG
	R: TCTGAAGGATTCTGGGGACA
*STAT5A*	F: GCAGAGTCCGTGACAGAGG
	R: CCACAGGTAGGGACAGAGTCT
*P53*	F: CCAGCAGCTCCTACACCGGC
	R: GAAACCGTAGCTGCCCTG
*NOS2*	F: ACTACAGGCTCGTGCAGGACTC
	R: CCACCACTCGCTCCAGGATACC

### Intracellular ROS detection

Intracellular ROS levels were measured using an ROS detection kit (Beyotime Biotechnology) following the manufacturer’s instructions. DCFH-DA (5 μM; Beyotime Biotechnology) was added to the cells of different groups, and incubated in the dark at 37°C for 30 min. The cells were then washed three times with serum-free culture medium, and ROS production was analyzed by flow cytometry on the BD Accuri C6 flow cytometer (BD Biosciences, Franklin Lakes, USA). The green fluorescence (DCF) detected was then evaluated between 500 and 530 nm (in the FL-1 channel) and red fluorescence (HE) between 590 and 700 nm (excitation wavelength 488 nm, emission wavelength 530 nm in the FL-2 channels). Data were expressed as the percentage of fluorescent cells. The sample without DCFH-DA treatment, DHE staining and centrifugation was evaluated as a negative control. Gating was performed to exclude any debris.

### Co-immunoprecipitation assay

Whole-cell protein lysates were extracted using cell lysis buffer containing PMSF (Beyotime Biotechnology). Endogenous immunoprecipitation was performed by incubating cell lysates with anti-ZIPK antibody (1:500; 30656-1-AP; Proteintech) or anti-STAT5A antibody (1:500; 366459-1-Ig; Proteintech), using anti-IgG antibody (1:500; 30000-0-AP; Proteintech) as the negative control, supplemented with 50 μL of protein A/G Dynabeads (Thermo Fisher Scientific) at 4°C overnight. After extensive wash, the bound protein was detected by western blot analysis using the corresponding primary and HRP-conjugated secondary antibodies ( Table 1) at a dilution ratio of 1:1000.

### Immunofluorescence assay

HUVECs were seeded in confocal culture dishes (Thermo Fisher Scientific). After the cells reached the appropriate density, they were fixed with 4% paraformaldehyde for 15 min, and permeabilized with 0.3% Triton X-100 for 5 min. The cells were then incubated overnight at 4°C with anti-ZIPK (1:500; 30656-1-AP; Proteintech) or anti-STAT5A antibody (1:500; 66459-1-Ig; Proteintech). The next day, the cells were treated with Plus 488-goat anti-mouse IgG secondary antibody (1:500; RGAM002; Proteintech) and Plus 594-goat anti-rabbit IgG secondary antibody (1:500; RGAR004; Proteintech) in PBS for 1 h at room temperature. After three times wash with PBS, the cell nuclei were stained with 4′,6-diamidino-2-phenylindole (DAPI). Images were captured using a confocal fluorescence microscope (Leica, Wetzlar, Germany).

### shRNA, siRNA, and plasmid treatments

HUVECs were transiently transfected with a ZIPK overexpression plasmid (ZIPK-OE), small interfering RNAs (siRNAs), or short hairpin RNAs (shRNAs) using Lipofectamine 2000 (Invitrogen, Carlsbad, USA) according to the manufacturer’s instructions. The ratio of plasmid, siRNA, and shRNA to Lipofectamine 2000 reagent was 1 μg/1.2 μL. The ZIPK-OE, siRNAs and shRNAs used in this study were obtained from Sangon Biotech (Shanghai, China) and the sequence information of siRNAs, shRNAs, and negative controls is shown in Table 3.

**Table:** **Table 3** The sequences of siRNAs and shRNAs

Name	Sequence (5′→3′)
sh-STAT5A-1	Sense: GCGCTTTAGTGACTCAGAAAT
	Anti-sense: AATTTCTGACTAAAGCGCG
sh-STAT5A -2	Sense: GAGAATTCACAGTCCTGTT
	Anti-sense: CTCTTAGTGTCAGACGGA
si-ZIPK-1	Sense: GGAACGAGUUCAAGAACAUdTdT
	Anti-sense: AUGUUCUUGAACUCGUUCCdTdT
si-ZIPK-2	Sense: ACGACAUCUUCGAGAACAAdTdT
	Anti-sense: UUGUUCUCGAAGAUGUCGUdTdT
Negative control (NC)	Sense: UUCUCCGAACGUGUCACGUTT
	Anti-sense: ACGUGACACGUUCGGAGAATT

### Rat model

The animal experiment was approved by the Research Ethics Committee of Zhongshan Hospital, Fudan University (2022-118). All procedures adhered to the “Guidelines for the Care and Use of Laboratory Animals” of Zhongshan Hospital and the National Institutes of Health, USA (2011 Edition). Sprague-Dawley rats weighing 300–400 g were obtained from SLAC Laboratory Animal Co., Ltd. (Shanghai, China) and were randomly assigned to 4 groups. The control group (Con, *n* = 10) received a single intraperitoneal injection of citrate buffer (0.1 M, pH 4.5). The diabetes group (DM, *n* = 10) was given a high-sugar and high-fat diet and then administered a single intraperitoneal injection of streptozotocin (3 mg/kg) three weeks after nephrectomy. To investigate the role of ZIPK in diabetic glomerular vascular injury, the ZIPK inhibitor TC-DAPK6 (MedChemExpress, Monmouth Junction, USA) was co-administered with streptozotocin at doses of 600 nM and 1200 nM [19] in 2% dimethyl sulfoxide (DMSO) and phosphate-buffered saline solution with pH adjusted to 7.4. These rats were designated as the DM+TCDAPK6-1 group ( *n* = 10) and DM+TCDAPK6-2 group ( *n* = 10), respectively.

### Immunohistochemistry and evaluation of staining

Immunohistochemical sections were prepared from glomerular tissue and the aortic arch of Sprague-Dawley (SD) rats by harvesting the tissues under sterile conditions. The kidneys were promptly removed, and the glomeruli were micro-dissected. The aortic arch was excised, and both tissues were fixed in a fixative solution to preserve cellular structure. Subsequently, the tissues were embedded in paraffin, thinly sliced, and mounted on glass slides for staining and analysis. Immunohistochemistry was performed on 3-μm paraffin-embedded tissue sections using anti-ZIPK, anti-P53, and anti-NOS2 antibodies ( Table 1). Following dewaxing and antigen retrieval, the sections were incubated with the primary antibodies overnight at 4°C, washed, and incubated with corresponding HRP-conjugated secondary antibodies ( Table 1). Finally, sections were visualized using a detection kit (P0603; Beyotime Biotechnology). Protein positivity was evaluated based on nuclear and/or cytoplasmic immunoreactivity. The average percentage of positive cells was determined from a minimum of 5 random fields at ×400 magnification and categorized as 0 (< 5%), 1 (5%‒25%), 2 (26%‒50%), 3 (51%‒75%), or 4 (> 75%). Staining intensity was graded as 1+ (weak), 2+ (moderate), or 3+ (intense). The positive cells score was calculated by multiplying the percentage and intensity, with scores ≥ 2 indicating positivity. Two independent pathologists who were blinded to the clinical data assessed the results.

### Structure prediction

AlphaFold3 [20] was run through its public web server ( https://www.alphafoldserver.com). Models were ranked based on AlphaFold’s standard model confidence score, and interface pLDDT (I-pLDDT) score was calculated as an additional confidence metric for each model, based on AlphaFold’s residue-level confidence scores (pLDDT) for residues at the TCR-pMHC interface.

### Statistical analysis

Statistical analyses were conducted using the SPSS 22.0 software package (IBM Corp., Armonk, USA). Student’s *t* test was used to compare two groups. For analyses involving more than two groups, one-way analysis of variance (ANOVA) and the *Chi*-squared test were employed. The results are presented as the mean±standard deviation (SD), and *P* < 0.05 was considered to indicate statistical significance.

## Results

### High glucose induces the upregulation of STAT5A in HUVECs, leading to increased expression of P53 and elevated ROS level

**Figure 1:**
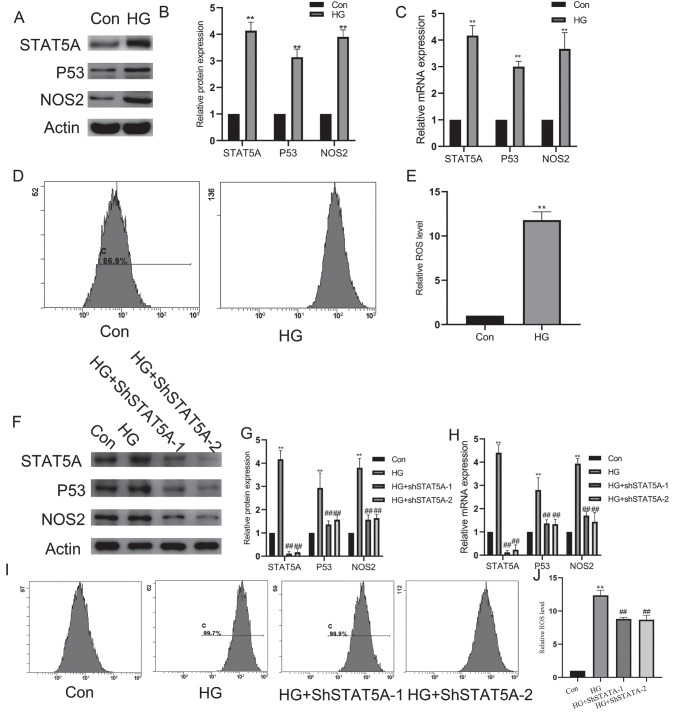
Figure 1 HG induces the upregulation of STAT5A, P53, and ROS levels in HUVECs (A) Western blot analysis of STAT5A, P53, and NOS2 protein levels following high glucose treatment. (B) Statistical analysis of protein expression levels (n = 3 per group). (C) qPCR analysis of the mRNA expression levels of STAT5A , P53, and NOS2 after the indicated treatments (n = 5 per group). (D) Flow cytometry analysis of intracellular ROS levels after 24 h. (E) Statistical analysis of ROS levels ( n=3 per group). (F) Western blot analysis was performed to assess STAT5A, P53, and NOS2 protein levels after exposure to high glucose and STAT5A silencing. (G) Statistical analysis of protein expression levels (n = 3 per group). (H) qPCR analysis of the mRNA expression levels of STAT5A, P53, and NOS2 after the indicated treatments (n = 5 per group). (I) Flow cytometry analysis of intracellular ROS levels after 24 h. (J) Statistical analysis of ROS levels (n = 3 per group). ** P < 0.01 vs the control group.

The upregulation of P53 is a critical mechanism and key biomarker for vascular endothelial damage caused by high blood sugar and diabetes. P53 also serves as an important molecular target in the treatment of diabetes. To determine the role of STAT5A in the high glucose-induced upregulation of P53 level and endothelial damage, HUVECs were cultured in normal glucose (Con, 5 mM, 6 days) and high glucose (HG, 25 mM, 6 days) media. Our results showed that high glucose induced the increased expressions of STAT5A, P53 and NOS2 at both the protein and mRNA levels in HUVECs ( Figure 1A–C). ROS assays revealed the accumulation of ROS and cell damage induced by high glucose in HUVECs ( Figure 1D,E). To elucidate the role of STAT5A in regulating the upregulation of P53 level and endothelial damage in high glucose-treated HUVECs, two independent shRNAs targeting *STAT5A* were used. Western blot analysis ( Figure 1F,G) and quantitative real-time PCR ( Figure 1H) confirmed that shSTAT5A promoted vascular endothelial ROS accumulation and the corresponding damage induced by high glucose ( Figure 1I,J). Silencing of *STAT5A* reduced the expression of P53 and NOS2 induced by high glucose ( Figure 1F–H). These data suggested that STAT5A positively regulates the expressions of P53 and NOS2, thereby mediating endothelial damage in high glucose-treated HUVECs.

### ZIPK is involved in the upregulation of P53 expression and ROS accumulation in HUVECs induced by high glucose

**Figure 2:**
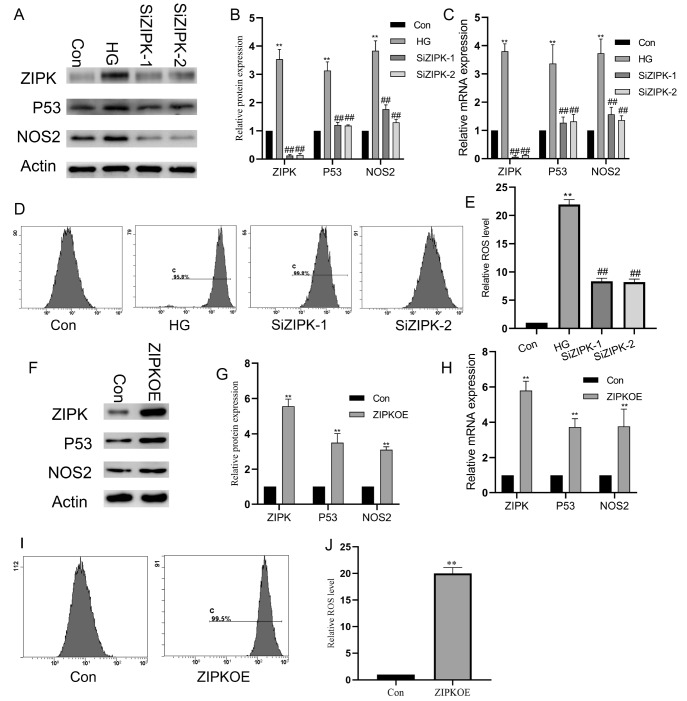
Figure 2 ZIPK is involved in the expressions of P53 and ROS accumulation in HUVECs induced by high glucose (A) Western blot analysis of STAT5A, P53, and NOS2 protein levels following high glucose treatment and reduced STAT5A expression. (B) Statistical analysis of protein expression levels (n = 3 per group). (C) qPCR detection of the mRNA expression levels of STAT5A, P53, and NOS2 under the indicated conditions (n = 5 per group). (D) Flow cytometry detection of intracellular ROS levels over 24 h in the experimental groups. (E) Statistical analysis of ROS levels (n = 3 per group). **P < 0.01 vs the control group, ## P < 0.01 vs the HG group. HG, high glucose.

Under high-glucose conditions, the expression of ZIPK was upregulated in a pattern consistent with that of P53 or NOS2 ( Figure 2A–C). To investigate the role of ZIPK in the high glucose-induced increase in P53 levels and endothelial damage, both loss- and gain-of-function approaches were used. Silencing of *ZIPK* counteracted the high glucose-induced expressions of P53 and NOS2 ( Figure 2A–C) and attenuated the accumulation of ROS and cell damage mediated by high glucose ( Figure 2D,E). Additionally, ZIPK overexpression mimicked high glucose treatment, upregulating the expressions of P53 and NOS2 in HUVECs ( Figure 2F–H) and promoting the accumulation of ROS ( Figure 2 I,J).

### ZIPK participates in the upregulation of P53 expression and ROS levels in HUVECs induced by high glucose through STAT5A

**Figure 3:**
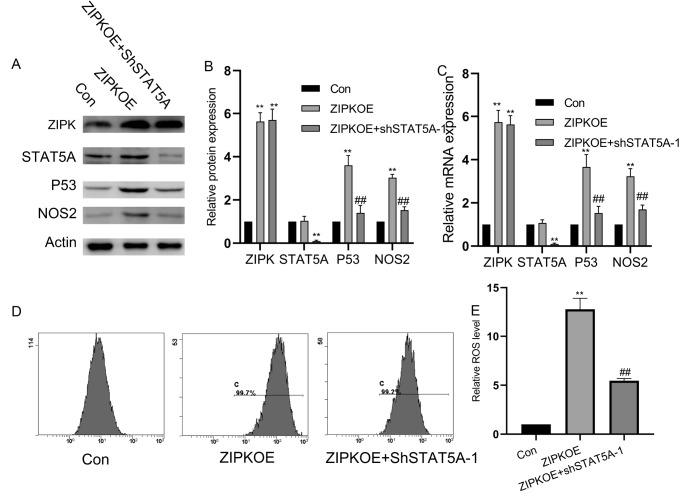
Figure 3 ZIPK is involved in the increase in P53 expression and ROS levels in HUVECs induced by high glucose through STAT5A (A) Western blot analysis of ZIPK, STAT5A, P53, and NOS2 protein levels following ZIPK overexpression and reduced STAT5A expression. (B) Statistical analysis of protein expression levels (n = 3 per group). (C) qPCR detection of the mRNA expression levels of STAT5A, P53, and NOS2 under the indicated conditions (n = 5 per group). (D) Flow cytometry detection of intracellular ROS levels over 24 h in the experimental groups. (E) Statistical analysis of ROS levels (n = 3 per group). **P < 0.01 vs the control group, ## P < 0.01 vs the ZIPK-OE group.

To explore whether ZIPK overexpression regulates the expression of STAT5A, we silenced *STAT5A* in ZIPK-overexpressing HUVECs. Downregulation of STAT5A expression reversed the induction of P53 and NOS2 expressions induced by ZIPK overexpression ( Figure 3A–C) and inhibited cellular ROS accumulation ( Figure 3D,E). These data suggested that ZIPK overexpression promotes the upregulation of P53 and NOS2 expressions in high glucose-treated HUVECs through STAT5A, thereby mediating the excessive accumulation of cellular ROS and cell damage.

### ZIPK interacts with STAT5A

**Figure 4:**
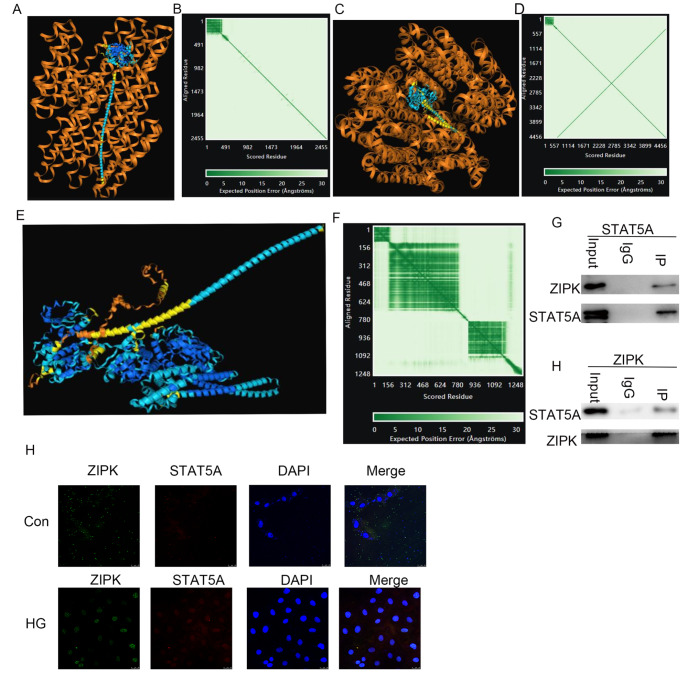
Figure 4 ZIPK interacts with STAT5A (A) Simulated diagram using AlphaFold3 showing the binding of ZIPK to the single-stranded DNA of the STAT5A promoter region. (B) Analysis of AlphaFold3 showing the binding of ZIPK to the single-stranded DNA of the STAT5A promoter region, with an interface-predicted template model (ipTM) = 0.19 and a predicted template model (pTM) = 0.28. (C) Simulated diagram using AlphaFold3 illustrating the binding of ZIPK to the double-stranded DNA of the STAT5A promoter region. (D) Analysis of AlphaFold3 demonstrating the binding of ZIPK to the double-stranded DNA of the STAT5A promoter region, with ipTM = 0.27 and pTM = 0.31. (E) Simulated diagram using AlphaFold3 showing the interaction between ZIPK and STAT5A. (F) Analysis of AlphaFold3 revealed an interaction between ZIPK and STAT5A, with ipTM = 0.16 and pTM = 0.49. (G) The interaction between ZIPK and STAT5A in HUVECs was determined by immunoprecipitation. (H) Confocal microscopy confirmed the colocalization of ZIPK and STAT5A in HUVECs.

To explore potential regulatory mechanisms, AlphaFold3 was used to predict the likelihood of ZIPK binding to the promoter region of *STAT5A* ( Figure 4A–D) and the likelihood of direct protein-protein interactions between ZIPK and STAT5A ( Figure 4E,F). Although the predictions were not conclusive, they suggested a greater likelihood of direct protein-protein interactions between the two proteins. Co-immunoprecipitation confirmed an interaction between the two proteins ( Figure 4G), and immunofluorescence co-localization experiments suggested that the proteins co-localized in the cell nucleus under high glucose stimulation ( Figure 4H).

### Inhibition of ZIPK in diabetic animal models and its effect on the expressions of endothelial cell-related proteins in renal glomeruli and aortic endothelial tissue

**Figure 5:**
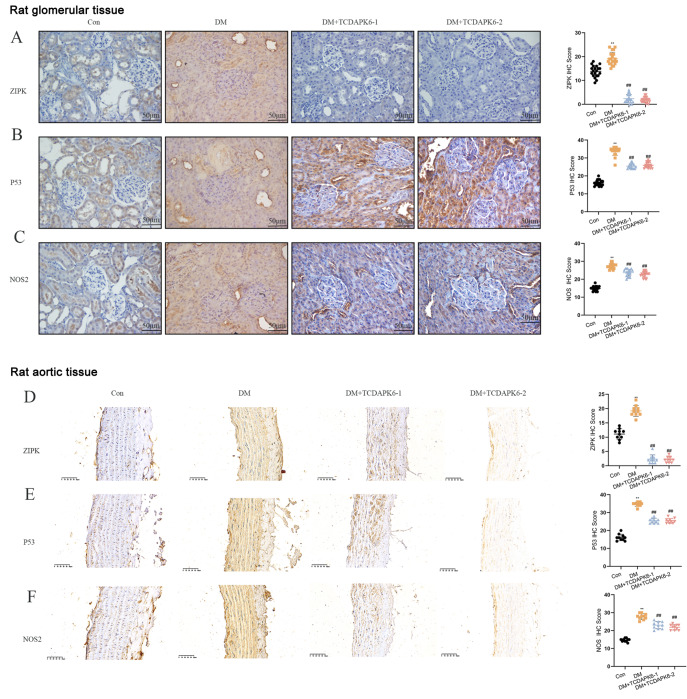
Figure 5 Inhibition of ZIPK expression in glomerular endothelial cells in diabetic animals (A) ZIPK expression in glomerular endothelial cells was detected by immunohistochemistry (n = 20 per group) in the renal glomeruli from both kidneys of each rat. (B) P53 expression in glomerular endothelial cells detected by immunohistochemistry ( n = 20 per group) in renal glomeruli from both kidneys of each rat. (C) NOS2 expression in glomerular endothelial cells detected by immunohistochemistry (n = 20 per group) in renal glomeruli from both kidneys of each rat. (D) ZIPK expression in glomerular endothelial cells detected by immunohistochemistry (n = 10 per group) in aortic endothelial tissue. (E) P53 expression in glomerular endothelial cells detected by immunohistochemistry (n = 10 per group) in aortic endothelial tissue. (F) NOS2 expression in glomerular endothelial cells detected by immunohistochemistry (n = 10 per group) in aortic endothelial tissue. *P < 0.01 vs the control group, ## P < 0.01 vs the DM group.

To confirm the role of ZIPK in vascular damage in diabetes, diabetic mice were successfully generated, and the ZIPK inhibitor TC-DAPK 6 was injected and validated through immunostaining. The results indicated that under high glucose stimulation, ZIPK, P53, and NOS2 were highly expressed, while inhibition of ZIPK led to decreased expressions of P53 and NOS2 in both renal glomeruli ( Figure 5A–C) and aortic endothelial tissues ( Figure 5D–F). These results confirmed the involvement of ZIPK in the high glucose-induced upregulation of P53 and vascular endothelial damage.

## Discussion

Diabetes mellitus is a chronic metabolic disorder characterized by hyperglycemia, leading to severe complications, including vascular damage. Vascular complications are a major cause of morbidity and mortality in diabetic patients and contribute to conditions such as retinopathy, nephropathy, and cardiovascular diseases [21]. The high-glucose environment in diabetes induces oxidative stress and inflammation, which are key factors in the pathogenesis of vascular damage [ 22, 23]. Understanding the molecular mechanisms underlying diabetes-induced vascular injury is crucial for developing effective therapeutic strategies.

In this study, we investigated the role of ZIPK in diabetes-related vascular damage. Previous research has shown that ZIPK is involved in various cellular processes, including apoptosis and inflammation [24]. Our findings indicated that high-glucose conditions significantly increased the expressions of ZIPK, STAT5A, P53, and NOS2 in HUVECs, suggesting that ZIPK plays a critical role in mediating oxidative stress and inflammatory responses in diabetic vascular injury. The use of TC-DAPK6 in a diabetic rat model demonstrated a protective effect against glomerular vascular damage, highlighting the potential of targeting ZIPK as a therapeutic approach for diabetic vascular complications [25].

ZIPK, also known as DAPK3, is a serine/threonine kinase that is crucial for apoptosis, autophagy, and cytoskeletal reorganization. High glucose significantly upregulates ZIPK expression in HUVECs, which is associated with increased P53 and NOS2 levels and elevated ROS accumulation [26]. The inhibition of ZIPK with TC-DAPK6 reduced glomerular endothelial cell damage in diabetic rats, underscoring the involvement of this kinase in diabetic vascular complications. Furthermore, STAT5A, a transcription factor, also shows elevated expression under high glucose conditions, promoting P53 and NOS2 expressions and ROS accumulation [27]. Silencing of *STAT5A* mitigates these effects, indicating its pivotal role in hyperglycemia-induced vascular damage. The interaction between ZIPK and STAT5A, confirmed by co-immunoprecipitation and immunofluorescence, suggested that ZIPK facilitates the transcriptional activation of P53 and NOS2 by STAT5A.

P53, a tumor suppressor, and NOS2, an enzyme responsible for nitric oxide production, are critical for oxidative stress and inflammation [28]. The upregulation of these genes under high-glucose conditions is mediated by the ZIPK-STAT5A axis, implicating this pathway in diabetic vascular injury. Inhibition of ZIPK also decreases NOS2 expression and oxidative stress *in vivo*, suggesting that ZIPK has therapeutic potential for diabetic vascular complications.

ROSs are highly reactive molecules that play dual roles in cellular physiology, acting as signaling molecules at low concentrations but causing oxidative damage at high concentrations [29]. In our study, we observed a significant increase in ROS levels in HUVECs exposed to high-glucose conditions, which was associated with elevated expressions of ZIPK, STAT5A, P53, and NOS2. The accumulation of ROS under hyperglycemic conditions leads to increased cellular damage and dysfunction, highlighting the role of oxidative stress in diabetic vascular complications. Silencing of *ZIPK* or *STAT5A* effectively reduced ROS levels, suggesting that the ZIPK-STAT5A signaling axis is a key regulator of oxidative stress in endothelial cells. These findings are consistent with previous reports that oxidative stress plays a central role in the pathogenesis of diabetic complications, including endothelial dysfunction and vascular damage.

In our study, we observed that high-glucose conditions significantly upregulated the expressions of ZIPK, STAT5A, P53, and NOS2 in HUVECs, leading to increased ROS levels and subsequent cellular damage. This finding aligns with previous research indicating that oxidative stress is a critical factor in diabetic vascular complications [30, 31]. The upregulation of STAT5A under high glucose conditions is a critical event, significantly reducing the expressions of P53 and NOS2 and attenuating ROS accumulation, thus preventing cell damage. This finding suggests that STAT5A plays a crucial role in mediating high glucose-induced vascular damage through the regulation of P53 and NOS2.

Furthermore, our data indicate that ZIPK is a key regulator of this pathway. ZIPK overexpression enhanced the high glucose-induced expressions of P53 and NOS2, while *ZIPK* silencing reversed these effects and reduced ROS levels. This finding is consistent with the notion that ZIPK may act upstream of P53 and NOS2 in the oxidative stress response pathway [14]. Moreover, our immunoprecipitation and immunofluorescence co-localization experiments demonstrated a direct interaction between ZIPK and STAT5A, with both proteins co-localizing in the nucleus under high-glucose conditions. This interaction suggests that ZIPK may facilitate the nuclear translocation of STAT5A, thereby promoting the transcriptional activation of P53 and NOS2.

*In vivo*, the use of TC-DAPK6 in diabetic rat models resulted in a significant reduction in the expressions of ZIPK, P53, and NOS2 in glomerular endothelial cells and aortic endothelial tissue, corroborating our *in vitro* findings. This indicates that ZIPK inhibition could be a potential therapeutic strategy for mitigating diabetic vascular damage, which highlights the therapeutic potential of targeting this kinase in diabetic complications.

Despite the significant findings, this study has several limitations that should be acknowledged. First, the study primarily relies on *in vitro* experiments using HUVECs and an *in vivo* rat model, which may not fully replicate the complexity of human diabetic vascular injury. Second, the sample sizes of both the cellular and animal experiments were relatively small, which may limit the generalizability of the results. Third, the study lacked clinical validation, which is crucial for translating these findings into potential therapeutic strategies for diabetic patients. Additionally, the use of multiple datasets and experimental conditions might introduce batch effects, which could affect the reproducibility and consistency of the results.

Future research should focus on validating these results in clinical settings and exploring the underlying mechanisms in detail. Longitudinal studies involving larger cohorts and diverse populations are essential to confirm the therapeutic potential of ZIPK inhibition. Additionally, investigating the interplay between ZIPK and other signaling pathways could provide a more comprehensive understanding of the molecular mechanisms driving diabetic vascular damage. Exploring the potential for combination therapies targeting multiple pathways simultaneously could also yield more effective treatment strategies.

In conclusion, our study demonstrated that ZIPK plays a significant role in diabetic vascular injury under high glucose conditions by modulating key signaling pathways involving STAT5A, P53, and NOS2. Our findings suggested that ZIPK could be a potential therapeutic target for mitigating vascular complications in diabetes patients.
